# Role of Environment and Experimenter in Reproducibility of Behavioral Studies With Laboratory Mice

**DOI:** 10.3389/fnbeh.2022.835444

**Published:** 2022-02-18

**Authors:** Martina Nigri, Johanna Åhlgren, David P. Wolfer, Vootele Voikar

**Affiliations:** ^1^Faculty of Medicine, Institute of Anatomy, University of Zurich, Zurich, Switzerland; ^2^Department of Health Sciences and Technology, Institute of Human Movement Sciences and Sport, ETH Zürich, Zurich, Switzerland; ^3^Laboratory Animal Center, HiLIFE, University of Helsinki, Helsinki, Finland; ^4^Neuroscience Center, HiLIFE, University of Helsinki, Helsinki, Finland

**Keywords:** mouse behavioral phenotyping, inbred strains, reproducibility, experimenter effect, environment effect

## Abstract

Behavioral phenotyping of mice has received a great deal of attention during the past three decades. However, there is still a pressing need to understand the variability caused by environmental and biological factors, human interference, and poorly standardized experimental protocols. The inconsistency of results is often attributed to the inter-individual difference between the experimenters and environmental conditions. The present work aims to dissect the combined influence of the experimenter and the environment on the detection of behavioral traits in two inbred strains most commonly used in behavioral genetics due to their contrasting phenotypes, the C57BL/6J and DBA/2J mice. To this purpose, the elevated O-maze, the open field with object, the accelerating rotarod and the Barnes maze tests were performed by two experimenters in two diverse laboratory environments. Our findings confirm the well-characterized behavioral differences between these strains in exploratory behavior, motor performance, learning and memory. Moreover, the results demonstrate how the experimenter and the environment influence the behavioral tests with a variable-dependent effect, often with mutually exclusive contributions. In this context, our study highlights how both the experimenter and the environment can have an impact on the strain effect size without altering the direction of the conclusions. Importantly, the general agreement on the results is reached by converging evidence from multiple measures addressing the same trait. In conclusion, the present work elucidates the contribution of both the experimenter and the laboratory environment in the intricate field of reproducibility in mouse behavioral phenotyping.

## Introduction

Behavior, representing the final output of the nervous system in all living organisms, results from the interaction between genotype and environment. Measures of behavioral outcomes are therefore essential for characterizing the animal models of neurodegenerative and neuropsychiatric diseases. As a consequence, behavioral phenotyping of genetically modified mice has turned to be a commonly used approach in behavioral neuroscience and genetics over the last 25 years (Voikar, 2020).

Along with the widespread use of this approach, some serious concerns about the validity and interpretation of data derived from knockout mice in general were raised, related to the problems with defining the genetic background of mutant mice (Gerlai, 1996; Silva et al., 1997). In addition, it appeared that conflicting results from different laboratories using supposedly the same mutant (or inbred) mouse lines were rather common and solution was seen in standardization.

In order to test the success of standardization, a seminal study was carried out in three laboratories (Crabbe et al., 1999). Despite rigorous standardization of test protocols, equipment, animals and many environmental variables, the outcome revealed systematic differences between the laboratories. Moreover, and more importantly, some phenotypic differences were dependent on the specific testing lab. These findings opened the debate over the need and usefulness of standardization (Würbel, 2000, 2002; Wahlsten, 2001; Van der Staay and Steckler, 2002) and in a way, paved the way to more extensive discussions about reproducibility (Editorial, 2009, 2013). Revisiting the 1999 study and provision of detailed analysis, revealed that the most salient difference between the laboratories might have been introduced by the persons having contact with the experimental animals (Wahlsten et al., 2003). The role of experimenter effect has been further addressed and confirmed by other studies (Lariviere et al., 2001; Chesler et al., 2002; Bohlen et al., 2014; Sorge et al., 2014). The method of handling of animals deserves also full appreciation (Hurst and West, 2010).

Another conclusion of extended analysis was that even if there were advantages of test standardization, the laboratory environments could never be made sufficiently similar to guarantee identical results (Wahlsten et al., 2003). In fact, for many assays achieving “identical” result is not needed – more important measure for reproducibility is to reach the consensus in the direction of the effect (Goodman et al., 2016; Kafkafi et al., 2018). However, this may not be possible to discuss or assess if the design and reporting of animal studies is deficient (Kilkenny et al., 2009; Editorial, 2019). To this end, the authors should familiarize themselves with guidelines for preparing, conducting and reporting before even starting the experiments (Smith et al., 2018; Percie du Sert et al., 2020). In addition, for sound and rigorous research, confirmation studies by different groups and coordinated multicenter trials are recommended (Mogil and Macleod, 2017).

Several multi-laboratory studies have been carried out since 1999. For instance, Lewejohann et al. concluded that the reliability of behavioral phenotyping is not challenged seriously by experimenter and laboratory environment as long as appropriate standardizations are met and suitable controls are involved (Lewejohann et al., 2006). In addition, development of standard operating procedures for large-scale phenotyping project generated reproducible results between laboratories for a number of the test output parameters (Mandillo et al., 2008). Another study demonstrated that analysis of mouse timing behavior led to robust and reliable endophenotypes across different labs (Maggi et al., 2014). Yet one more project addressed the standardization of experimental conditions in multi-laboratory effort (Richter et al., 2011). Overall, these studies recognize the need for good planning and expertise in behavioral testing as a prerequisite for reliable and reproducible research. It would be important to add here that even more reproducible results have been obtained when animals are studied by means of automated home-cage based approach (Krackow et al., 2010; Robinson and Riedel, 2014; Robinson et al., 2018; Arroyo-Araujo et al., 2019). On the other hand, such automated and unbiased measurements are still able to detect differences in behavior between the laboratories, which may need to be considered in evaluation (Pernold et al., 2019).

The availability of well-characterized inbred mouse strains allows investigators to study the gene-environment interactions. Efforts are made toward establishing ‘mouse phenome’ database where reference values of common inbred strains in a variety of behavioral tasks and physiological measurements can be found (Paigen and Eppig, 2000; Moldin et al., 2001). The C57BL/6 and DBA/2J mice are the oldest, and probably the most commonly used inbred strains in behavioral genetics. For many behavioral domains, they are considered to display a moderate phenotype (Crawley et al., 1997), which allows a feasible detection of behavioral changes at the baseline and in response to various manipulations (Stiedl et al., 1999; Cabib et al., 2000; Voikar et al., 2005; Youn et al., 2012).

The aim of the present study was to further evaluate the relative impact of the experimenter and the environment on replicability of mouse behavioral phenotype. To this aim, a battery of behavioral test was performed by two experimenters in two diverse laboratory environments. Selection of behavioral tests was based on the assumption that both objective (automated recording by video-tracking) and subjective (handling, manually recorded behavior) measures were considered. The C57BL/6 and DBA/2J inbred strains were deliberately chosen for their markedly different and well-characterized behaviors. However, no particular emphasis was placed on standardizing environmental parameters.

## Materials and Methods

All the behavioral tests were carried out by a 25-year-old female experimenter (M) and a 49-year-old male experimenter (V) in two diverse laboratory environments: the Institute of Anatomy in Zürich (Z) and the Laboratory Animal Center in Helsinki (H). All the experimental procedures were carried out in accordance with the European legislation (Directive 2010/63/EU), having been approved by the veterinary office of the Canton of Zürich (license number 060/2021) and National Animal Experiment Board of Finland (license ESAVI/10165/04.10.07/2016).

### Animals and Environment

Four batches of eight weeks old female C57BL/6J (*n* = 12) and DBA/2J (*n* = 12) mice were obtained from Charles River Laboratories (France). Thus, the total number of animals used was 96 (48 C57BL/6J and 48 DBA/2J). The mice were kept in same strain-groups of 4 in standard Type III cages (ZH: temperature 21.9 ± 0.3°C and relative humidity 60.2 ± 9.6%) or in individually ventilated cages (HE: temperature 21.7 ± 0.4°C and relative humidity 55.5 ± 5.3%) for an adaptation period of three weeks before the behavioral testing. Food and water were available *ad libitum* (see the Table 1 and Supplementary Figure 3, for details). Cage changes occurred once a week since the mice arrived at the testing animal facility. Before testing, the animal caretakers took care of clean cages. To avoid stress during behavioral testing, cage changes were always performed on Fridays, allowing animals to adapt to new cages over the weekend. During the experiment (starting with handling, marking and weighing the mice), the experimenter taking care of the entire behavioral test battery was also moving the mice to the clean cages. The first two batches were housed under a 12/12 inverted light-dark cycle (light on 20:00–8:00) and the testing occurred during the dark phase in Zurich (in August 2018). The mice in Helsinki (third and fourth batch) were exposed to normal light (light on 6:00–18:00) with the behavioral testing occurring during the light phase (in September 2018).

**TABLE 1:** Details of housing and husbandry in two laboratories.

	Zurich	Helsinki
Light cycle	Reversed (light on 20:00–8:00)	Normal (light on 6:00–18:00)
Food	KLIBA NAFAG – Switzerland; Aliment for mice and rats – 3436 (pellet 15 mm)	Envigo Global Diet 2916C (pellet 12 mm)
Water	Tap water, *ad libitum*	Filtered and UV-irradiated, *ad libitum*
Bedding	Aspen chips 2.5–3.5 mm; J.RETTENMAIER & SÖHNE GMBH + CO KG; Rosenberg, Germany	aspen chips 5 mm × 5 mm × 1 mm, 4HP; Tapvei, Estonia
Nest material	Tissue paper	aspen strips, PM90L, Tapvei, Estonia
Additional enrichment	Red plastic shelter (Zoonlab); cardboard shelter	3 aspen bricks (50 mm × 10 mm × 10 mm, Tapvei, Estonia)
Cage	Eurostandard Type III cage, dimensions 425 mm × 276 mm × 153 mm, floor area 820 cm^2^; covered with filter top; Tecniplast, Italy	Mouse IVC Green Line – overall cage dimensions 391 mm × 199 mm × 160 mm, floor area 501 cm^2^; Tecniplast, Italy
Cage change	once/week	once/week
Temperature (measured during exp)	21.9 ± 0.3°C (mean, SEM)	21.7 ± 0.4°C (mean, SEM)
Humidity (measured during exp)	60.2 ± 9.6% (mean, SEM)	55.5 ± 5.3% (mean, SEM)
Animal facility	Conventional	Standard Pathogen Free
Protecting clothing	Disposable cap and coat on top of personal clothing, lab shoes, gloves	Full re-dressing - cap, mask, coat, socks, lab shoes, gloves, entry to animal facility through air shower
Time of experiments	Between 8:30 and 15:00; 20.7.-10.8.2018	Between 8:30 and 15:00; 31.8.-28.9.2018

### Video Tracking

During the elevated O-maze, open field with object and Barnes maze tests, the mice were video tracked using a Noldus Ethovision XT15 system (Noldus Information Technology, Wageningen, The Netherlands). The data were exported to custom designed software Wintrack (Wolfer et al., 2001) for further analysis.

### Conventional Behavioral Testing

**FIGURE 1:**
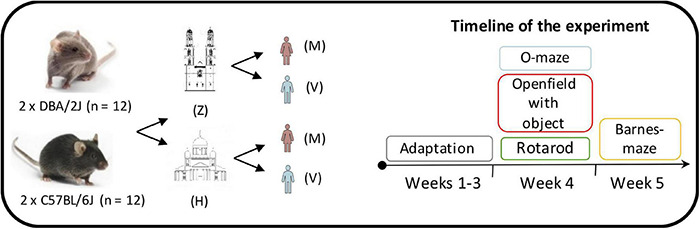
Overview of the experimental design. The behavioral testing was carried out using two batches of eight weeks old C57BL/6J (*n* = 12) and DBA/2J (*n* = 12) mice. All the behavioral procedures were applied by a 25-year-old female experimenter (M) and a 49-year-old male experimenter (V) in two different laboratories: the Institute of Anatomy in Zürich (Z) and the Laboratory Animal Center in Helsinki (H). An adaptation phase of four weeks was followed by the elevated O-maze, open field with object, rotarod and Barnes maze tests.

The behavioral testing started when the mice were 12 weeks old and each experimenter was introduced to them by a gentle handling (∼3 min – picking up from the cage, tail marking, measuring body weight, and allowing to explore on the experimenter’s palm) three days before start of testing. Sample size calculation was based on previous experience. Same protocols and similar testing procedures were applied by two experimenters in the two laboratories. The behavioral tests were carried out in the following order: elevated O-maze, open field with object, rotarod and Barnes maze tests. Order of testing the animals was randomized and counterbalanced. A schematic overview of the experimental approach is presented in Figure 1.

### Behavioral Procedures

#### Elevated O-Maze

The test is used to assess unconditioned anxiety like-behaviors in mice (Shepherd et al., 1994). The behavioral device consists of a 5.5 cm wide annular runway with an outer diameter of 46 cm. The apparatus was placed inside the large open field arena approximately 40 cm above the floor. The two opposing 90°closed sectors are protected by 16 cm high inner and outer walls of grey polyvinyl chloride. The remaining two open sectors (30 × 5 cm) have no walls. Illumination was applied by indirect diffuse room light (20–25 lux). During the experiment the animals were placed in the center of the maze facing one of the closed sectors and observed for 10 min. Exploratory head dips, stretched attends, grooming and rearing events were manually recorded using the keyboard event-recorder provided by the video tracking system.

#### Open Field With Object

The test is used to measure locomotion, anxiety, explorative and stereotypical behaviors such as grooming and rearing in rodents (Walsh and Cummins, 1976; Voikar and Stanford, 2021). The behavioral apparatus consisted of four 50 cm × 50 cm arenas (with wall height of 40 cm) placed under camera for recording. The illumination was applied by indirect diffuse room light (20–25 lux). Each animal was released in one of the corners and monitored for 15 min. The mice were then removed and placed in the holding cage, the number of the fecal boli was counted and a 12 cm × 4 cm semi-transparent 50 ml falcon tube was placed in the center of each arena. The animals were then released in the arena and observed for additional 15 min.

#### Rotarod

Motor coordination and learning was tested by using the digitally controlled mouse rotarod apparatus (Ugo Basile, Italy). The device has a drum with diameter of 30 mm and provides adjustable speed (2–80 rpm) and acceleration (6″–600″). The illumination was applied by indirect diffuse room light (20–25 lux). Four mice were simultaneously placed on the rotarod apparatus with the rod rotating at 4 rpm during the first minute. The rotation speed is increased every 30 s by 4rpm and a trial terminates either when the mouse falls down or when 5 min are completed. Each animal was submitted to five trials with an inter-trial interval of 30 min. The time to fall, digitally and manually recorded, provides the measure of motor ability and the improvement across trials measures the motor learning.

#### Barnes Maze

The test is used to assess spatial learning and memory in mice and rats (Barnes, 1979). The maze consists of a circular platform (100 cm diameter) with 20 holes (5 cm diameter) around the perimeter (Ugo Basile, Italy). One of the holes was connected with a dark chamber filled with bedding material and two food pellets, the escape box. Two days before the experiment, each animal was introduced to the escape box for 2–3 min. The bright light (500–600 lux on the platform) was used to induce the mice to find and enter the escape box. The mice were trained to find the escape box in three training trails per day (inter-trial interval at least 60 min) over three days. The training trial ended when the mouse entered the escape box or after 3 min as cut-off time (in this case, the mouse was gently directed to the escape box). The memory test was carried out during the first trial on day 4 when the mice were monitored on the platform without escape box for 90 s. Thereafter, reversal learning was carried out, where the escape box was moved under the opposite hole and the mice received three training trials on day 4 and 5. After the last training trial on day 5, the second memory test was performed.

### Statistical Analyses

The statistical analysis, blinded and performed by a third person, was conducted using an ANOVA model with strain (B6 = C57BL/6J, D2 = DBA/2J), experimenter (M = female experimenter, V = male experimenter) and laboratory environments (Z = Zürich, H = Helsinki) as between subject factors. Significant interactions were further explored by pairwise t-tests or by splitting the ANOVA model, as appropriate. Variables with strongly skewed distributions or strong correlations between variances and group means were subjected to Box-Cox transformation before the statistical analysis. The significance threshold was set at 0.05 and the false discovery rate (FDR) control procedure of Hochberg was applied to groups of conceptually related variables within single tests to correct significance thresholds for multiple comparisons. Cohen’s *d* was used as measure of the size of strain differences, partial omega squared as measure of the size of ANOVA effects and interactions. Pooled data of the four experiments was additionally analyzed using Bayesian statistics (R package “BayesFactor”), permitting to probe the data not only for presence but also for absence of a strain effect (Keysers et al., 2020). Precisely, a Bayes factor (BF) was computed as the likelihood ratio between alternative models with and without strain effect, given the observed data. A BF > 3 was taken as moderate evidence for, a BF < 1/3 as moderate evidence against presence of a strain effect. BF > 10 and BF < 1/10 were interpreted as strong evidence for and against a strain effect, respectively. The pooled data as pseudo-population permitted to tentatively identify false positive (positive test outcome despite evidence for absence of a strain effect in the pseudo-population) and false negative results (negative test outcome despite evidence for presence of a strain effect in the pseudo-population) in individual experiments. The statistical analyses and graphs were obtained using R version 4.1.2, complemented with the packages “effectsize” and “ggplot2.” In bar and line graphs, untransformed data are plotted as mean + SEM with individual data points shown in the background.

## Results

### Phenotypic Profile of C57BL/6J and DBA/2J Mice

**FIGURE 2:**
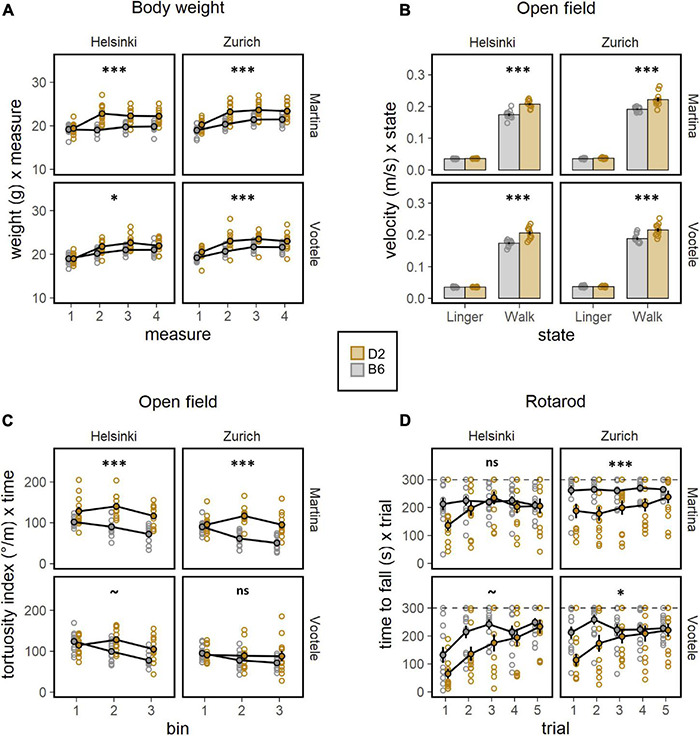
Results of the behavioral battery of tests. **(A)** Body weight (g) during the behavioral testing (ANOVA: strain F1,88 = 57.46, *p* < 0.0001, ω^2^ = 0.40, measure F3,264 = 133.0, *p* < 0.0001, ω^2^ = 0.60). DBA/2J mice were much heavier than C57BL/6J mice and weight gain was more sustained in C57BL/6J than DBA/2J mice. The strain effect was detected by both experimenters and in both laboratories (*post-hoc* test: *p < 0.05, ***p < 0.001 for strain effect). **(B)** Lingering (m/s) defined as sum of resting and any deceleration and walking velocity (m/s) in the open field (ANOVA: strain F1,88 = 74.48, *p* < 0.0001, ω^2^ = 0.46, strain × state F1,88 = 36.38, *p* < 0.0001, ω^2^ = 0.29). DBA/2J mice displayed higher walking velocity compared to C57BL/6J mice. The strain effect was detected by both experimenters and in both laboratories (*post-hoc* test: ***p < 0.001 for strain effect). **(C)** Tortuosity index defined as sum of unsigned direction changes divided by total distance moved in the openfield (ANOVA: strain F1,88 = 36.52, *p* < 0.0001, ω^2^ = 0.29). DBA/2J showed an higher tortuosity index compared to C57BL/6J mice. The strain effect was detected in 2 of 4 individual experiments: missed in VZ and VH (*post-hoc* test: ***p < 0.001, ~*p* < 0.1 for strain effect). **(D)** Time to fall (s) as measure of motor learning and coordination ability in the rotarod (ANOVA: strain F1,88 = 18.44, *p* < 0.0001, ω^2^ = 0.17, trial F1,376 = 49.97, *p* < 0.0001, ω^2^ = 0.12, strain × trial F1,376 = 17.90, *p* < 0.0001, ω^2^ = 0.05). Overall, performance improved across trials in the rotarod indicating motor learning with the DBA/2J mice falling earlier during the initial phase of testing. The main effect of strain was missed when the mice were tested in Helsinki (*post-hoc* test: *p < 0.5, ***p < 0.001, ~*p* < 0.1 for strain effect).

**FIGURE 3:**
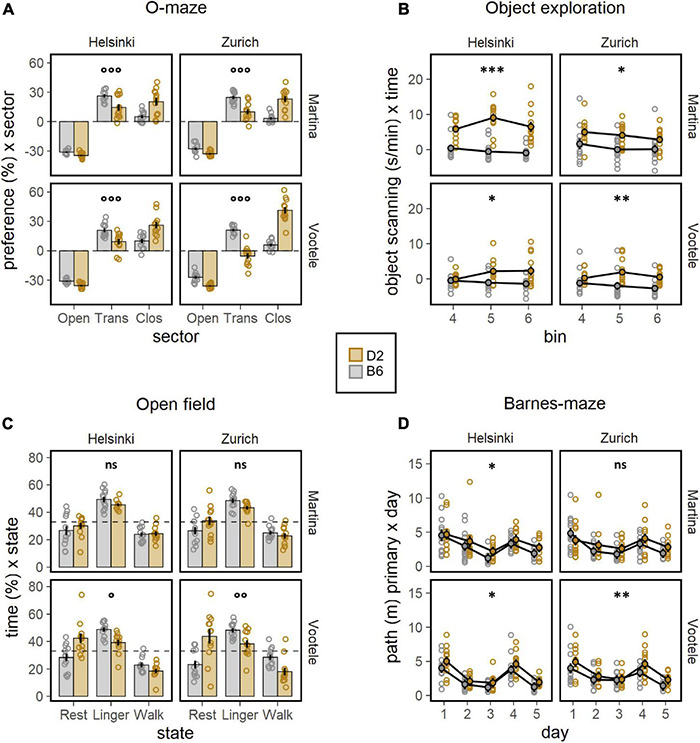
Results of the behavioral battery of tests. **(A)** Preference for sector (%) in the elevated O-maze (ANOVA: strain × sector F1,184 = 28.02, *p* < 0.0001, ω^2^ = 0.13). DBA/2J mice showed much stronger avoidance of open sectors and less preference for the transition zones, in favor of a much stronger preference for the closed sectors. This was detected by both experimenters in both laboratories (*post-hoc*-test: °°°*p* < 0.001 for strain × sector interaction). **(B)** Object scanning (m/min) as measure of exploratory activity in the open field with object (ANOVA: strain F1,88 = 48.45, *p* < 0.0001, ω^2^ = 0.36). DBA/2J spent more time exploring the object in the open field with object whereas C57BL/6J showed the strain-typical absence of object exploration. This was detected by both experimenters in both laboratories (*post-hoc*-test: *p < 0.05, **p < 0.01, ***p < 0.001 for strain effect). **(C)** Percentage of time (%) × state as measure of the motor profile in the open field (ANOVA: strain × state F1,184 = 12.79, *p* = 0.0004, ω^2^ = 0.06). DBA/2J mice displayed higher resting time percentage and lower percentage of walking time in the open field. This was detected in 2 of 4 individual experiments: missed in MZ and MH (*post-hoc* test: °*p* < 0.05, °°*p* < 0.01 for strain × state interaction). **(D)** Distance moved (m) as measure of spatial learning abilities in the Barnes maze (ANOVA: strain F1,88 = 51.44, *p* < 0.0001, ω^2^ = 0.37, day F4,352 = 21.92, *p* < 0.0001, ω^2^ = 0.20). Overall, distance moved to find the escape hole showed a robust learning, reversal and re-learning effect, indicating that the protocol worked as intended. DBA/2J moved a longer distance to find the escape hole in the Barnes maze. The strain effect was missed in MZ experiment (*post-hoc* test: *p < 0.05, **p < 0.01 for strain effect).

To deeply investigate the well documented behavioral differences between C57BL/6J and DBA/2J mice, a battery of behavioral tests was performed by both experimenters (M, V) in both laboratory environments (Z, H, Figures 2, 3). The body weight of mice, measured before and during the behavioral testing, revealed a significant strain effect with DBA/2J showing a higher body weight than C57BL/6J mice (F1,88 = 57.46, *p* < 0.0001, Figure 2A). Moreover, the weight gain was more pronounced in C57BL/6J than in DBA/2J mice during the behavioral testing (F3,264 = 16.10, *p* < 0.0001, Figure 2A). Specifically looking at the locomotor activity and coordination ability, the significant main effects of strain on locomotion revealed how DBA/2J mice displayed higher walking velocity (F1,88 = 74.48, *p* < 0.0001, ω^2^ = 0.46, Figure 2B) combined with a higher tortuosity index (F1,88 = 36.52, *p* < 0.0001, Figure 2C) in the open field. Overall, performance improved across trials in the rotarod indicating motor learning with the DBA/2J mice falling earlier during the initial phase of testing (F1,376 = 17.90, *p* < 0.0001, Figure 2D). These data indicated DBA/2J being characterized by a faster and less linear locomotion combined with a poorer coordination. To address anxiety like behaviors in C57BL/6J and DBA/2J mice, the elevated O-maze test was performed by both experimenters in both laboratories. Results elucidated a much stronger avoidance of open sectors and less preference for transition zones, in favor of a much stronger preference for closed sectors in DBA/2J compared to the C57BL/6J mice (F1,184 = 28.02, *p* < 0.0001, Figure 3A). This was also confirmed in the open field test where DBA/2J mice showed much stronger avoidance of center zone in favor of a much stronger preference for the transition and wall zones (strain × zone F2,176 = 60.01, *p* < 0.0001, ω^2^ = 0.41, Supplementary Figure 4B). In addition, C57BL/6J showed the strain-typical absence of object exploration in the open field with object whereas DBA/2J mice spent more time exploring the object without sign of habituation (F1,88 = 48.45, *p* < 0.0001, Figure 3B). Focusing on the motor profile, the main effect of strain on activity revealed how DBA/2J mice displayed higher resting time percentage and lower percentage of walking time in the open field (F1,184 = 12.79, *p* = 0.0004, Figure 3C). Overall, distance moved to find the escape hole showed a robust learning, reversal and re-learning effect in the Barnes maze test performed by both experimenters in both laboratories. Interestingly, DBA/2J mice moved a longer distance to find the escape hole (F1,88 = 51.44, *p* < 0.0001, Figure 3D) taking longer time to finding it (F1,88 = 34.16, *p* < 0.0001, Supplementary Figure 4C) indicating worse spatial learning abilities. Remarkably, our data detected the notorious strain behavioral differences between C57BL/6J and DBA/2J mice.

### Experiments Agree Over Direction of Effect

**FIGURE 4:**
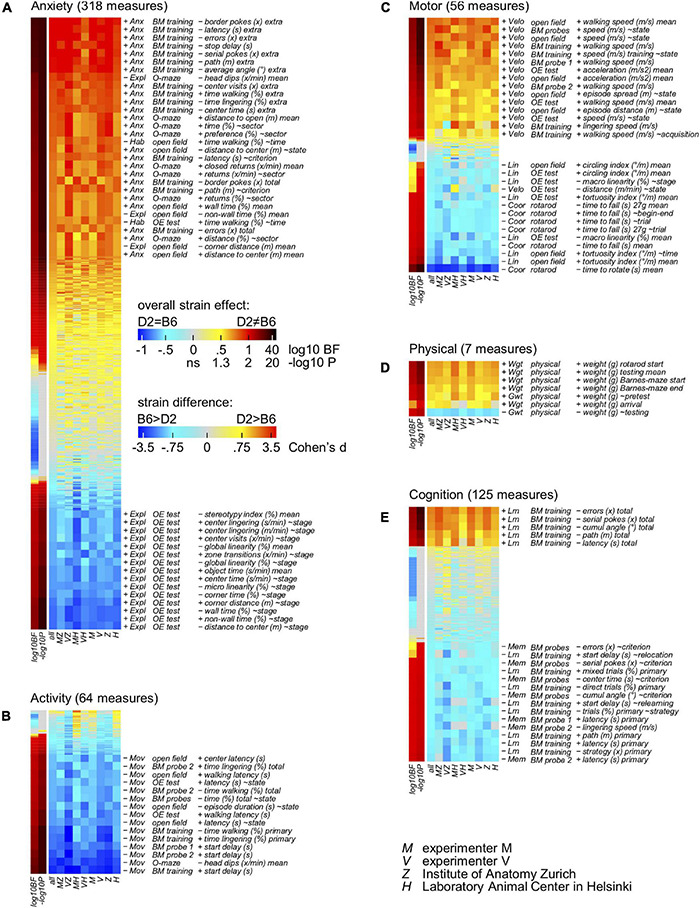
Heatmaps indicating the direction of the strain effect. Behavioral measures with multiple observations per animal (repeated measures) were converted to factorial measures by taking the average across observations (205 mean of selected repetitions) or by computing the slope across observations (321 slope across selected repetitions). All the 526 behavioral variables have a primary assignment to a behavioral domain; 44 variables have a secondary assignment in addition. Open field and OE test slope variables (∼time: bin 1-2-3, 5 min each, ∼stage: open field-OE test, ∼zone: (prospective) object-transition-wall, ∼direction: centripetal-fugal, ∼state: rest-linger-walk); BM training and probe slope variables (∼acquisition: day 1-2-3, ∼relearning: day 1-4-5, ∼relocation: day (3| 5)-4, ∼state: rest-linger-walk, ∼criterion: primary-extra, ∼strategy: mixed-serial-direct, ∼place: control-target, ∼angle: 72-54-36-18-0° deviation); physical slope variables (∼testing: time during behavioral testing, ∼pretest: arrival to begin of testing); rotarod slope variables (∼trial 1-2-3-4-5, ∼begin-end 1-5); O-maze slope variables (∼time: bin 1-2, 5 min each, ∼sector: open-transition-closed, ∼position: free-protected). Individual columns show effects obtained by individual experiments, persons and labs with the second lane indicating the *p*-value of the overall ANOVA strain effect. In addition, experiments were analyzed using a pseudo population approach also with Bayesian stats permitting to obtain evidence for absence of effect. **(A)** Overview of 318 anxiety related measures, sorted by overall Cohen’s *d* as measure of size of the strain effect in a strain × person × lab ANOVA model. Measures related to exploration were treated as negative measures of anxiety and included in the table after multiplying d with –1. DBA/2J mice earned higher scores on measures of anxiety and lower scores on measures of exploration. **(B)** Overview of 64 activity related measures, sorted by overall Cohen’s *d* as measure of size of the strain effect in a strain × person × lab ANOVA model. Measures related to resting and lingering were treated as negative measures of activity and included in the table after multiplying d with –1. DBA/2J mice moved less and later, earning lower scores on measures of activity and higher scores on measures of inactivity. **(C)** Overview of 56 motor related measures, sorted by overall Cohen’s *d* as measure of size of the strain effect in a strain × person × lab ANOVA model. Locomotion of DBA/2J mice was characterized by faster walking as well as less linear and less predictable trajectories combined with coordination deficit. **(D)** Overview of 7 physical related measures, sorted by overall Cohen’s *d* as measure of size of the strain effect in a strain × person × lab ANOVA model. DBA/2J mice showed higher body weight but gained less weight during the behavioral testing. **(E)** Overview of 125 cognition related measures, sorted by overall Cohen’s *d* as measure of size of the strain effect in a strain × person × lab ANOVA model. Error scores were treated as negative measures of learning performance and included in the table after multiplying d with –1. DBA/2J mice earned poor scores of spatial selectivity during training and probe trials on the Barnes maze.

To deeply examine the consistency of the direction of the observed strain differences obtained by the two experimenters in the two laboratories, a novel statistical approach based on the analysis of multiple tests addressing a single behavioral domain was developed. To this end, all the 526 measures (Supplementary Table 1) obtained from the behavioral experiments were assigned to at least one behavioral domain: physical, motor, anxiety, activity and cognition. All the measures related to each behavioral domain were then sorted by overall Cohen’s *d* as measure of size of the strain effect in a strain × person × lab ANOVA model and heatmaps were generated accordingly (Figure 4). Using our novel approach and looking specifically at the anxiety domain (Figure 4A), results agreed on the direction of the strain effect with DBA/2J obtaining higher scores on measures of anxiety and lower scores on measures of exploration and habituation. This was most evident in the Barnes maze where many measures reflect the fact that DBA/2J mice disappeared more rapidly after having found the escape box. In this context, wall-related measures in the open field test yield the largest strain effects since DBA/2J mice avoided both the center and the transition zones more than C57BL/6J mice. Due to the notorious avoidance reaction of C57BL/6J in the test, scores of object exploration show a reversal pattern. Looking at the activity profile of C57BL/6J and DBA/2J mice, the heatmap presented in Figure 4B confirmed a good agreement on the direction of the strain effect with DBA/2J mice collecting lower scores on measures of activity. Precisely, data confirmed how they moved less and later. While the strain effect on latency related variables and head dips may be boosted by their increased anxiety, distance related measures tended to show smaller effects due to their increased speed of locomotion. Agreement in the context of the motor profile related measures was also achieved (Figure 4C). In this context, the heatmap revealed DBA/2J to be characterized by a faster and less linear locomotion combined with coordination deficit. The less predictable trajectories observed in DBA/2J mice were less pronounced in the open field test due to their increased wall preference. Specifically looking at the rotarod related measures, their performance was poor with a very strong tendency to hold and rotate on the drum instead of walking on it. General agreement was also obtained in the context of physical related measures with DBA/2J mice showing higher body weight but gaining less weight during the testing compared to C57BL/6J mice (Figure 4D). Focusing on the cognitive profile of C57BL/6J and DBA/2J mice, agreement on the direction of the strain effect in learning and memory abilities was reached (Figure 4E). In this context, data showed how DBA/2J mice earned poor scores of spatial selectivity during training and probe trials on the Barnes maze. This would also imply higher error scores which was counteracted by their generally reduced locomotor activity. Surprisingly and in light of the mentioned results, data suggested how experiments agree over direction of the strain effect.

### Each Single Experiment Agrees With the Others

**FIGURE 5:**
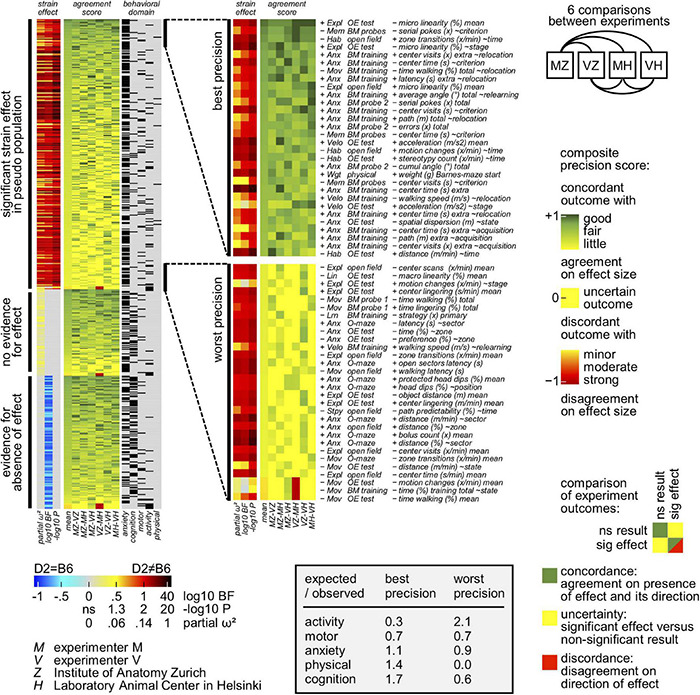
Results showing reproducibility between experiments. All possible six comparisons between individual experiments (MZ, MH, VZ, VH) have been made, as presented in the heatmaps. According to agreement on both the presence and the direction of the strain effect, three outcome categories are obtained: concordance, uncertainty and discordance. A score of 1 was assigned when concordant effects with identical size based on the Cohens’ *d* were observed. In contrast, discordant effects obtained a score of –1. False positives appear less frequent than false negatives, hence more non-concordance when there is an effect in the pseudo population – without evident relationship between size of the strain effect and rate of non-concordance. In addition, learning and memory related measures were strongly overrepresented in the subset of the most reproducible measures. In contrast, measures of activity and size determined by video tracking are overrepresented in the subset of the least reproducible measures.

The reproducibility of each single experiment was then deeply investigated. To evaluate how well one single experiment agrees with the others in terms of strain effect direction of each behavioral measure, all possible six comparisons between individual experiments (MZ, MH, VZ, VH) have been made (Figure 5). According to agreement on both the presence and the direction of the strain effect, three outcome categories are obtained: concordance, uncertainty and discordance. The latter two are considered as failure of one experiment to replicate the other. In this context, a precision score was assigned to each behavioral measure based on the presence of either concordant or discordant effects. Precisely, a score of 1 was assigned when concordant effects with identical size based on the Cohens’ *d* were observed. In contrast, discordant effects obtained a score of −1. Surprisingly, 75% precision scores are > 0 indicating reproducible results, either true positives or true negatives. Additionally, and in agreement with the threshold of 5% set for type-I error, false positive results are 4.6%. Interestingly, our data elucidated how discordant strain effects are very few and mostly explained by the compromised detection of body size by the video tracking system. Remarkably, results highlighted how the strain effect was highly reproducible for all the behaviors tested.

### High Versus Low Reproducible Measures

The degree of reproducibility of variables belonging to each behavioral domain was then deeply evaluated. The previously mentioned approach based on the presence of either concordant or discordant effects was used, and precision scores were assigned accordingly. The heatmaps presented in Figure 5 elucidated how measures of learning and memory were strongly overrepresented in the subset of the most reproducible measures and to a lesser degree also motor performance related measures. Importantly, measures of activity determined by video tracking are overrepresented in the subset of the least reproducible measures. Interestingly, our data show object exploration and O-maze related parameters being overrepresented in the subset of the least reproducible measures. Importantly, our data were able to detect both the most and the least reproducible measures belonging to the addressed behavioral domains.

### Experimenter Impact on Size and Direction of the Strain Effect

**FIGURE 6:**
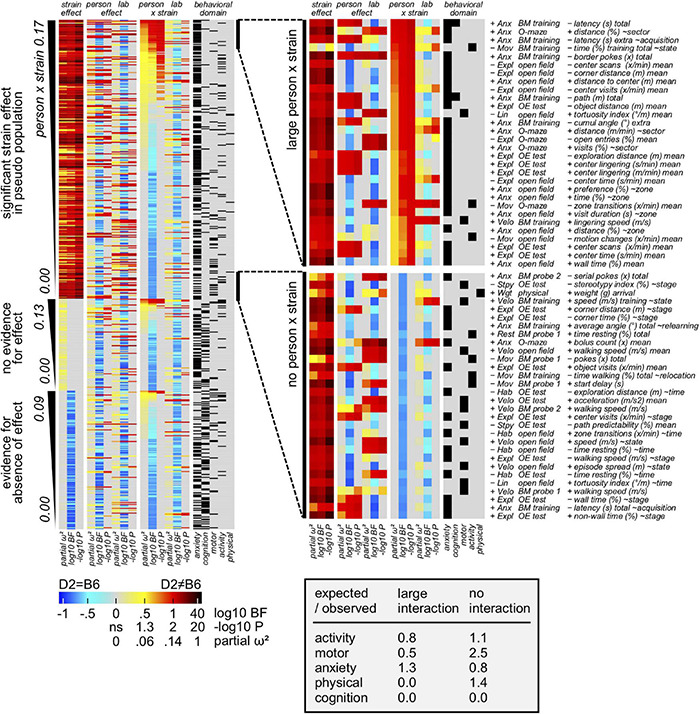
Results showing the experimenter contribution to overall variance. Behavioral measures assigned to 3 sections according to strain effect in pseudo population: evidence for, inconclusive, evidence against (false positives). Based on partial omega squared of the interaction, 30 variable subsets with overall strain effect and large vs small person × strain interactions, were extracted. A modest enrichment of anxiety measures in the subset with large person × strain interaction is observed. Additionally, motor related measures seem relatively resistant against person × strain interaction.

Having defined both the most and the least reproducible measures, we were then interested in deeply evaluating the relative influence of the experimenter on size/direction of the strain effect (experimenter × strain interaction). To this aim, behavioral measures were assigned to 3 sections according to strain effect in pseudo population: evidence for, inconclusive, evidence against. Precisely and based on partial omega squared of the interaction, 30 variable subsets with overall strain effect and large vs small experimenter × strain interactions, were extracted and analyzed. Heatmaps presented in Figure 6 elucidated how measures of motor performance are strongly overrepresented in the subset of the least affected measures. Interestingly, data showed measure of learning and memory as well as size being not present in the subset of the most affected measures. In contrast, both activity and anxiety related parameters appeared to be affected by the experimenter. Importantly, while the Barnes maze is overrepresented in the subset of least affected related measures, open field and object exploration are overrepresented in the subset of the most affected related parameters. Rotarod and physical examination, by contrast, do not contribute to the subset of the most affected measures. Interestingly, data presented in Supplementary Figure 1 showed how the impact of the experimenter on concordance is minor (5–10%) compared to the total strain effect size (70%).

### Environment Impact on Size and Direction of the Strain Effect

**FIGURE 7:**
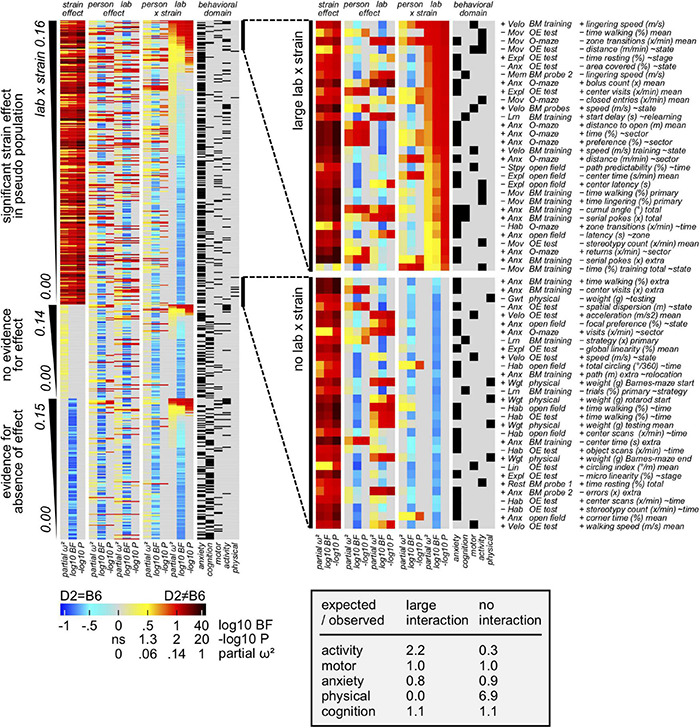
Results showing the environment contribution to overall variance. Behavioral measures assigned to 3 sections according to strain effect in pseudo population: evidence for, inconclusive, evidence against (false positives). Based on partial omega squared of the interaction, 30 variable subsets with overall strain effect and large vs small environment × strain interactions, were extracted. Importantly, results elucidated how enrichment of activity related measures in the subset with large lab × strain interactions was detected. In contrast, physical related parameters seem particularly resistant against lab × strain interaction.

The relative impact of the environment on size/direction of the strain effect (environment × strain interaction) was also investigated using the previously mentioned approach. In this context, results (Figure 7) elucidated measures of learning and memory as well as size determined by physical examination being the least affected by the laboratory environment. In contrast, both measures of activity and size determined by video tracking appeared to belong to the most affected measures. Importantly, learning and memory related parameters were not present in the subset of the most affected measures by the laboratory environment. Looking specifically at the O-maze and object exploration, results highlighted their related measures being influenced by the laboratory environment. Remarkably, our results elucidated how experimenter and environment effects are mutually exclusive and independent of strain effects. Considering the mentioned results, data presented in Supplementary Figure 2 showed the impact of the laboratory environment being similar to the experimenter one, accounting for a minor impact on concordance (5–10%) compared to the strain effect size (70%).

## Discussion

The reproducibility and replicability of the experimental work in biomedical research has been a hot topic fueling intensive debates during the past 10 years (Baker, 2016; Fitzpatrick et al., 2018). Indeed, irreproducibility prevalence rates have been estimated to range between 50 and 90% (Prinz et al., 2011; Begley and Ellis, 2012; Begley and Ioannidis, 2015). Several reasons for poor reproducibility have been identified – publication bias, inappropriate statistical analysis, lack of randomization and blinding, validation of reagents (Landis et al., 2012; Begley, 2013; Munafò et al., 2017). Recently concluded and published results of cancer reproducibility project highlights many of these issues (Editorial, 2021; Mullard, 2021). Working with animals requires consideration of many more issues which are critical for the validity of an experiment (Smith, 2020).

With the present study, our aim was to add information on the role of environment and experimenter in behavioral phenotyping of mouse models. Importantly, the aim was not to evaluate the standardization of the procedures. However, two laboratories had extensive experience (>25 years) in behavioral testing and had similar equipment available. Therefore, the “standardization” covered only agreement on the behavioral protocols, and the source (and strain) of animals used. Inbred strains provide an important tool for understanding genetic mechanisms underlying behavior. By large, the phenotypic differences between inbred strains are suggested to be stable over time and across laboratories, although the behaviors related to emotional, cognitive and social processes may be labile and affected by laboratory-specific parameters in husbandry and testing (Wahlsten et al., 2006). In order to investigate the experimenter and the environment contribution separately, our study deeply explored their relative impact on the detection of behavioral traits in C57BL/6J and DBA/2J female mice. Overall, this approach is in line with the concept of systematic heterogenization (Richter, 2017, 2020; Voelkl et al., 2020) recommended for enhancing external validity and generalization (Karp, 2018; Eggel and Wurbel, 2021).

Several previous studies have elucidated how the experimenter and the laboratory environment may account for the variation across replicate studies within or between laboratories (Chesler et al., 2002; Wahlsten et al., 2003; Bohlen et al., 2014). Considering that highly reproducible finding under highly standardized conditions may poorly generalize to other experimental conditions (lab or experimenter), the same protocols and similar testing procedures were applied in our study, consisting of four replications. This approach allowed us systematically collect data in large cohort of mice (pooled data as a pseudo-population) and thereafter, to focus on the measures of reliability and validity (precision and accuracy) of each replication (mini-experiments).

As expected, significant strain differences were revealed for each behavioral test. In accordance with previous findings, the DBA/2J mice were less active, showing enhanced anxiety-like behavior and avoidance of exposed areas (in open field and elevated O-maze) with impaired motor performance (on rotarod) when compared to C57BL/6J mice (Voikar et al., 2005; Kulesskaya and Voikar, 2014; Ahlgren and Voikar, 2019). In contrast, exploration of the novel object in the center of open field was enhanced in the DBA/2J mice as also shown in previous studies (Kim et al., 2005). In spatial learning and memory tasks, and fear conditioning, the DBA/2 mice have been usually described as inferior compared to the C57BL/6 strain (Crawley et al., 1997; Logue et al., 1997; Holmes et al., 2002; Youn et al., 2012). In line with earlier reports, the difference in spatial learning abilities between the two strains were also detected in our study.

Confirming the differences between the two strains allowed us deeply evaluate the relative impact of both the experimenter and the environment on the behavioral results. We developed and applied a novel statistical approach based on the analysis of multiple tests addressing a single behavioral domain. Interestingly, variable dependent effects of both the experimenter and the environment are detectable but not capable to alter the direction of conclusions. Surprisingly, main effects of the experimenter and the environment are mutually exclusive and remarkably good deal of consistency of the strain effect is observed. Importantly, accounting for 75% of the total variability, strain effect was highly reproducible for all the behaviors tested and importantly, well-documented strain differences were detected. Additionally, in agreement with the threshold of 5% set for type-I error, false positives are 4.6%.

Two major environmental differences between the laboratories were the phase of light cycle when the testing occurred and the housing system used. Alarmingly, up to 70% of publications fail in disclosing the circadian time when the animals are administered the treatment (Alitalo et al., 2021). Although testing during the dark period may be intuitively and ethologically more relevant, the fact is that many laboratories do not apply inverted light cycle because of various practical and logistic reasons. Moreover, for basic behavioral testing it has been shown that many parameters are not affected by the time of testing, and discriminate the strains well in the active or inactive period (Hossain et al., 2004; Beeler et al., 2006; Deacon, 2006; Yang et al., 2008; Robinson et al., 2018). Even if the differences depending on the time of testing (during light or dark phase) are detected, the comparison to the other studies is often complicated because of specific design (only male or female animals, single or group housed) or missing information on test conditions (Roedel et al., 2006; Richetto et al., 2019). Importantly, it is suggested that mice can adapt to the daily activity of laboratory personnel (Robinson-Junker et al., 2018). Taken together, the findings of all these previous experiments and our data can be summarized that if the differences between testing during light and dark phase exist, they may be heavily dependent on variety of factors (strain, sex, housing conditions, illumination during testing, the test situation) (Peirson et al., 2018). Additionally, little or no evidence is reported for impaired welfare or sleep deprivation when mice are disturbed for testing or husbandry procedures during the light phase (Robinson-Junker et al., 2018, 2019).

The individually ventilated cages (IVC) are becoming a mainstream housing condition for laboratory rodents. Although there are clear benefits for monitoring hygiene, microbiological status and importantly, health hazards for personnel, the impact on animal physiology and behavior has been extensively discussed. The data so far show that the changes in the phenotype of mice may be dependent on the parameters studied and laboratories (Mineur and Crusio, 2009; Logge et al., 2013; Ahlgren and Voikar, 2019). Based on our data, we suggest that neither light cycle nor housing system obscured the phenotypic differences between the C57BL/6 and DBA/2 mice.

Using only female mice in our study may be considered as a limitation. However, the main aim of this study was to investigate the impact of environment and experimenter in behavioral phenotyping experiments. Therefore, we planned it by employing two inbred strains, to identify the genotype × environment interactions. We did not include male mice because (1) the sex difference was not of major interest in this proof-of-principle study (including three factors – genotype, experimenter and laboratory) and (2) personal experience is that ordering male mice from the commercial supplier at the age of 6 weeks or later often results in fighting and need to single housing/re-grouping which may be a major drawback for the design and conduct of the study (Weber et al., 2017). In addition, convincing evidence exists that phenotypic variability may be higher in males than females and exact information on the phase of the estrous cycle is not necessary in basic studies with laboratory rodents (Prendergast et al., 2014; Becker et al., 2016; Fritz et al., 2017; Shansky and Murphy, 2021). Examining the influence of the estrous cycle on a particular experimental question is always an option, but is not required for research in females, just as assessing testosterone levels (which can vary up to tenfold across a cohort) is not a standard practice for experiments in males (Shansky, 2019). However, this should not be taken as underestimating the importance of sex differences in biomedical research (Karp et al., 2017; Breznik et al., 2021).

Testing animals in more than one laboratory in a coordinated preclinical trial can definitely support the reliability and generalization of findings. However, involving more than one laboratory requires certainly more attention on planning and logistics of the study. We have been partners in several such endeavors, which have produced a lot of useful data but also emphasizing how important is the coordination of the project, because many things can go wrong already before actual start of the experiments (Krackow et al., 2010; Richter et al., 2011; Codita et al., 2012). For instance, ordering the mice from commercial vendor may seem easy and straightforward, but it may appear that suddenly they do not have available mice at desired age, or in particular breeding facility. Therefore, planning checklists and culture of care (good communication) cannot be promoted enough (Smith, 2020; Robinson et al., 2021). Finally, we want emphasize experience and training for conducting behavioral experiments, because failure to consider essential factors affecting behavior of mice, interaction of mice and experimenters, and scoring behavior, may strongly influence the reproducibility, validity and reliability of the experiments (Blizard et al., 2007; Rodgers, 2007; Stanford, 2007; Schellinck et al., 2010; Voikar, 2020).

In summary, by applying novel statistical approach, we elucidated how large strain differences are robust and are unlikely to alter the direction of the behavioral results. Highlighting how reproducible results can be reached by converging evidence from multiple measures addressing the same behavioral domain, our work deeply examined the contribution of the experimenter and the environment and provided novel insights in the intricate field of behavioral phenotyping.

## Data Availability Statement

The original contributions presented in the study are included in the article/Supplementary Material, further inquiries can be directed to the corresponding authors.

## Ethics Statement

All the experimental procedures were carried out in accordance with the European legislation (Directive 2010/63/EU), having been approved by the veterinary office of the Canton of Zürich (license number 060/2021) and National Animal Experiment Board of Finland (license ESAVI/10165/04.10.07/2016).

## Author Contributions

MN, JÅ, DW, and VV: design and concept of the study, local support and coordination with planning, protocols, equipment and animal orders. MN and VV: mouse behavioral phenotyping. DW: statistical analysis. All authors discussed the data and wrote the manuscript.

## Conflict of Interest

The authors declare that the research was conducted in the absence of any commercial or financial relationships that could be construed as a potential conflict of interest.

## Publisher’s Note

All claims expressed in this article are solely those of the authors and do not necessarily represent those of their affiliated organizations, or those of the publisher, the editors and the reviewers. Any product that may be evaluated in this article, or claim that may be made by its manufacturer, is not guaranteed or endorsed by the publisher.

## Supplementary Material

Supplementary Figure 1 Results indicating the impact of person × strain interaction. Person × strain interactions are by definition expected to negatively impact on precision. Their impact in comparison to the effect of the size of the strain effect was examined and reported in the graphs. Person × strain interactions have a detectable impact on the measurement of effect size and a smaller one on the detection of presence and direction of effects. Importantly, the impact of person × strain interactions (5–10%) on concordance is minor compared to the impact of strain effect size or power (70%).

Supplementary Figure 2 Results indicating the impact of laboratory × strain interaction. Laboratory × strain interactions are by definition expected to negatively impact on precision. Their impact in comparison to the effect of the size of the strain effect was examined and reported in the graphs. Laboratory × strain interactions have a detectable impact on the measurement of effect size and a smaller one on the detection of presence and direction of effects. Large laboratory × stain interactions increase false negative as well as false positive rate and true discordance. Importantly, the impact of person × strain interactions (5–10%) on concordance is minor compared to the impact of strain effect size or power (70%).

Supplementary Figure 3 Details on the cage environment. **(A)** Cages equipped with two shelters (one cardboard and one red plastic, Zoonlab) and paper tissue as nesting material in Zürich. **(B)** Cages equipped with wooden gnawing blocks and abundant nesting material providing also a shelter (aspen strips) in Helsinki.

Supplementary Figure 4 Results of the behavioral battery of tests. **(A)** Distance moved (m) × time in the open field (ANOVA: bin F1,184 = 118.9, *p* < 0.0001, ω^2^ = 0.39, strain F1,88 = 1.398 ns, strain × bin F1,184 = 87.76, *p* < 0.0001, ω^2^ = 0.32). Overall, distance moved decreased with time indicating habituation. Additionally, no evidence for an overall strain effect was observed. DBA/2J mice moved less during the first 5 min of the experiment compared to C57BL/6J mice. This was detected by both experimenters in both laboratories (*post-hoc*-test: °°*p* < 0.01, °°°*p* < 0.001 for strain × bin interactions). **(B)** Preference × zone (%) in open field (ANOVA: strain × zone F2,176 = 60.01, *p* < 0.0001, ω^2^=0.41). DBA/2J mice showed much stronger avoidance of center zone in favor of a much stronger preference for the transition and wall zones. This was detected by both experimenters in both laboratories (*post-hoc*-test: °°*p* < 0.01, °°°*p* < 0.001 for strain × zone interactions). **(C)** Latency (s) primary × as measure of spatial learning abilities in the Barnes maze (ANOVA: strain F1,88 = 34.16, *p* < 0.0001, ω^2^ = 0.28, day F4,352 = 69.05, *p* < 0.0001, ω^2^ = 0.44). Overall, latency to find the escape hole showed a robust learning, reversal and re-learning effect, indicated the protocol worked as intended. DBA/2J mice took longer to find the escape hole. The strain effect was missed in MH and MZ experiments (*post-hoc* test: ^***^*p* < 0.001 for strain effect, ~*p* < 0.1).

Supplementary Table 1 List of the 526 behavioral related variables.
