# Supplementary figures and images for: Role of Environment and Experimenter in Reproducibility of Behavioral Studies With Laboratory Mice
Source: Front Behav Neurosci. 2022 Feb 18;16:835444. doi: 10.3389/fnbeh.2022.835444 (PMC8895324; doi:10.3389/fnbeh.2022.835444)

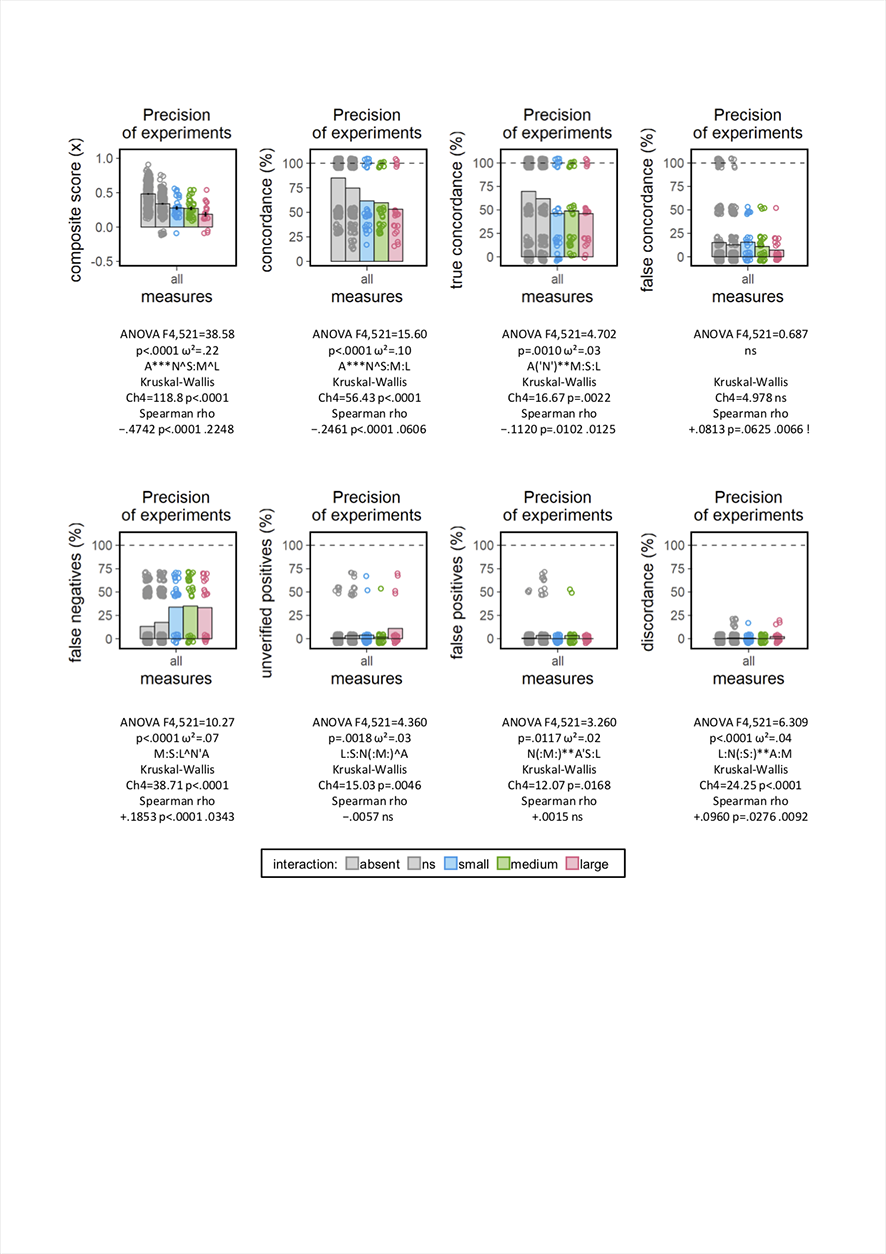

Supplement: Supplementary Figure 1 — Results indicating the impact of person × strain interaction. Person × strain interactions are by definition expected to negatively impact on precision. Their impact in comparison to the effect of the size of the strain effect was examined and reported in the graphs. Person × strain interactions have a detectable impact on the measurement of effect size and a smaller one on the detection of presence and direction of effects. Importantly, the impact of person × strain interactions (5–10%) on concordance is minor compared to the impact of strain effect size or power (70%). [file Image_1.TIFF]

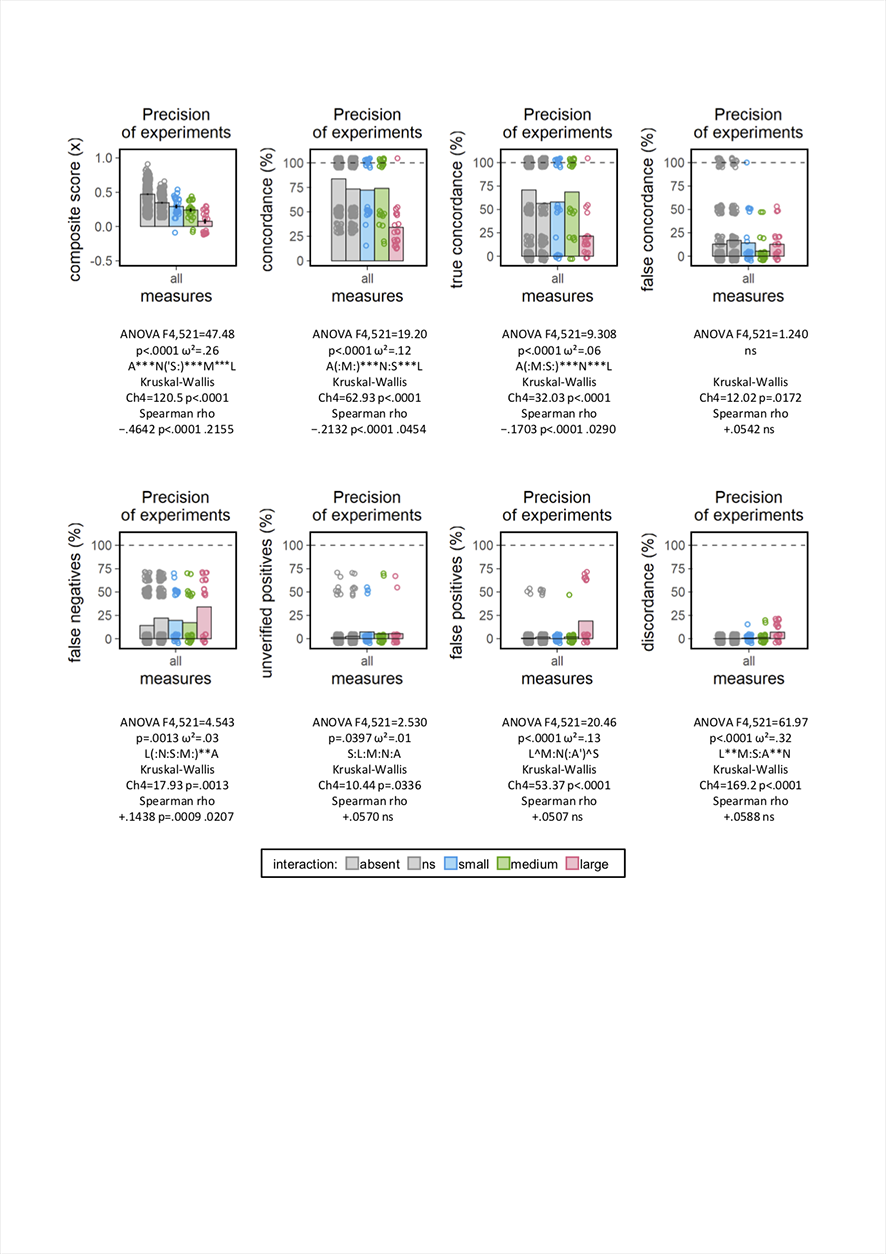

Supplement: Supplementary Figure 2 — Results indicating the impact of laboratory × strain interaction. Laboratory × strain interactions are by definition expected to negatively impact on precision. Their impact in comparison to the effect of the size of the strain effect was examined and reported in the graphs. Laboratory × strain interactions have a detectable impact on the measurement of effect size and a smaller one on the detection of presence and direction of effects. Large laboratory × stain interactions increase false negative as well as false positive rate and true discordance. Importantly, the impact of person × strain interactions (5–10%) on concordance is minor compared to the impact of strain effect size or power (70%). [file Image_2.TIFF]

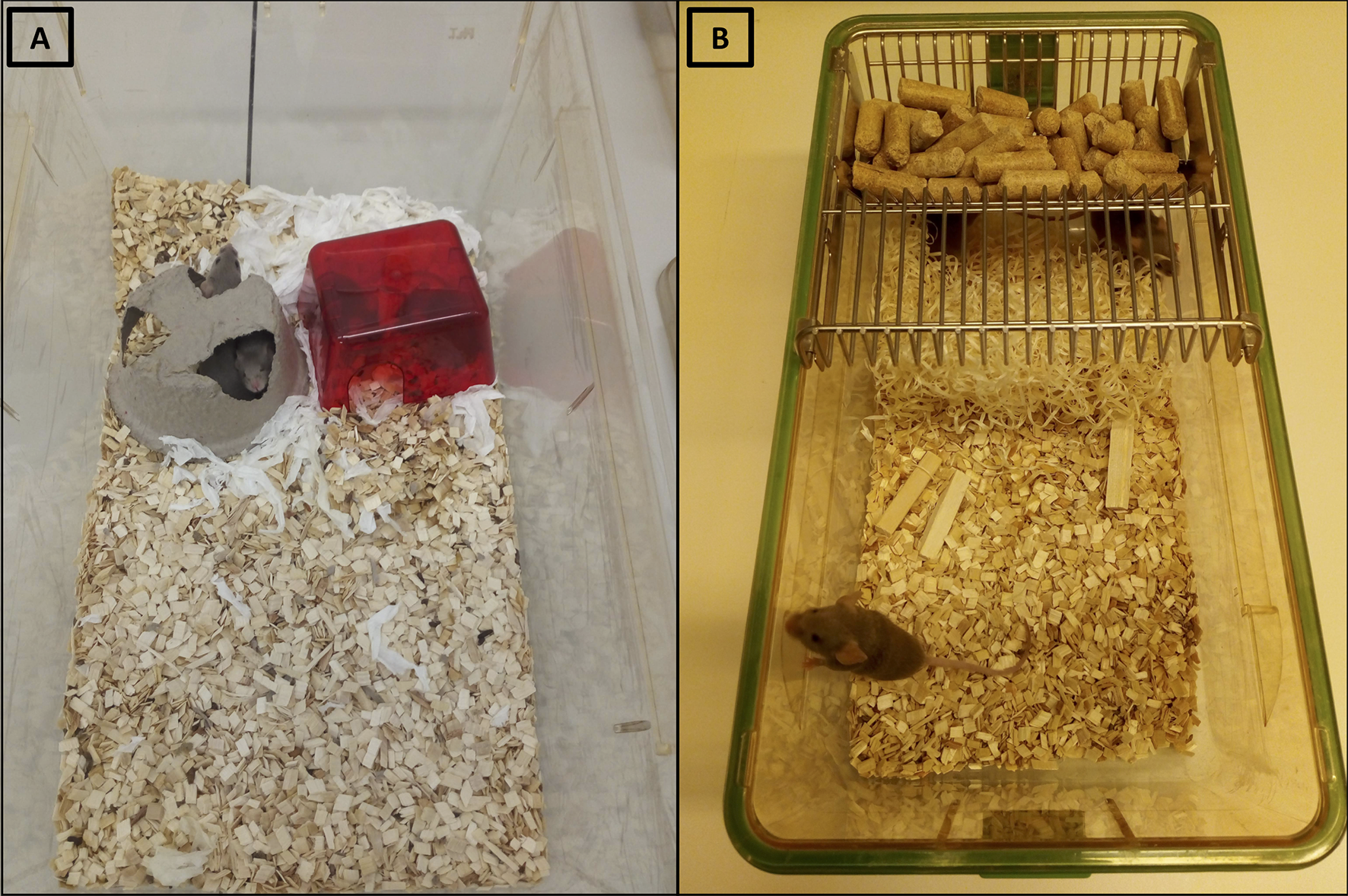

Supplement: Supplementary Figure 3 — Details on the cage environment. (A) Cages equipped with two shelters (one cardboard and one red plastic, Zoonlab) and paper tissue as nesting material in Zürich. (B) Cages equipped with wooden gnawing blocks and abundant nesting material providing also a shelter (aspen strips) in Helsinki. [file Image_3.TIFF]

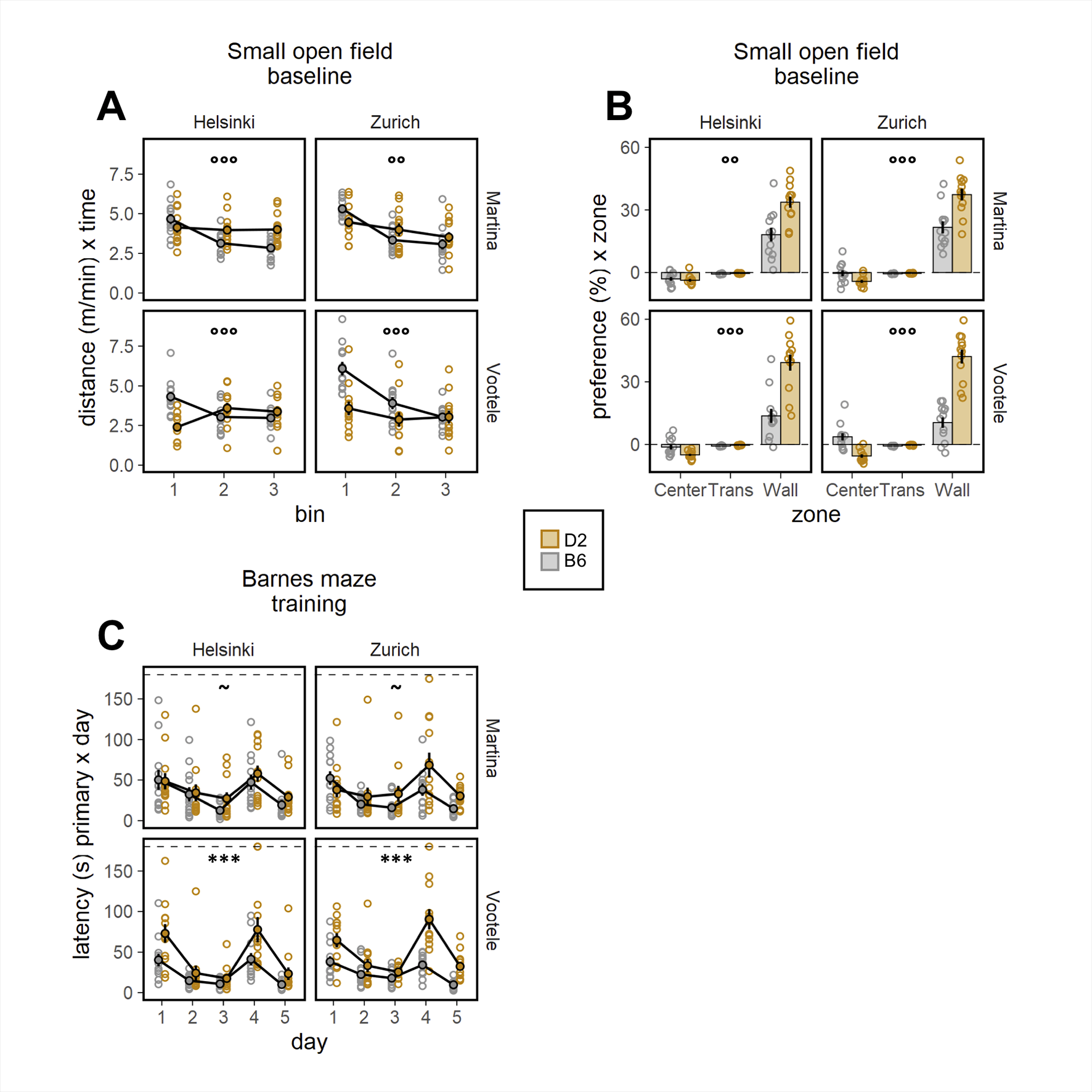

Supplement: Supplementary Figure 4 — Results of the behavioral battery of tests. (A) Distance moved (m) × time in the open field (ANOVA: bin F1,184 = 118.9, p < 0.0001, ω2 = 0.39, strain F1,88 = 1.398 ns, strain × bin F1,184 = 87.76, p < 0.0001, ω2 = 0.32). Overall, distance moved decreased with time indicating habituation. Additionally, no evidence for an overall strain effect was observed. DBA/2J mice moved less during the first 5 min of the experiment compared to C57BL/6J mice. This was detected by both experimenters in both laboratories (post-hoc-test: °°p < 0.01, °°°p < 0.001 for strain × bin interactions). (B) Preference × zone (%) in open field (ANOVA: strain × zone F2,176 = 60.01, p < 0.0001, ω2=0.41). DBA/2J mice showed much stronger avoidance of center zone in favor of a much stronger preference for the transition and wall zones. This was detected by both experimenters in both laboratories (post-hoc-test: °°p < 0.01, °°°p < 0.001 for strain × zone interactions). (C) Latency (s) primary × as measure of spatial learning abilities in the Barnes maze (ANOVA: strain F1,88 = 34.16, p < 0.0001, ω2 = 0.28, day F4,352 = 69.05, p < 0.0001, ω2 = 0.44). Overall, latency to find the escape hole showed a robust learning, reversal and re-learning effect, indicated the protocol worked as intended. DBA/2J mice took longer to find the escape hole. The strain effect was missed in MH and MZ experiments (post-hoc test: ***p < 0.001 for strain effect, ~p < 0.1). [file Image_4.TIFF]
